# Food Security and Nutrition as the Neglected Missing Links in Cultural Evolution: The Role of the Sociotype

**DOI:** 10.5041/RMMJ.10477

**Published:** 2022-07-31

**Authors:** Elliot M. Berry

**Affiliations:** Braun School of Public Health, Hebrew University–Hadassah Medical School, Jerusalem, Israel

**Keywords:** Complex adaptive systems, cultural evolution, food security, *Homo culturus*, Neolithic agricultural revolution, nutrition, sociotype

## Abstract

Food security and nutrition were major drivers of cultural evolution by enabling sociotypic development and communal living after the Neolithic agricultural revolution some 12,000 years ago. The sociotype unites concepts from the sciences and the humanities; in concert with the genotype it determines an individual’s phenotype (observable traits and behavior), and together they advance societal culture. As such, the sociotype relates to an individual’s dynamic interactions with the surrounding social environment throughout life and comprises three domains: the Individual, Relationships, and Context. Nutrition affects each domain, respectively, by ensuring the following dimensions of food security: utilization (metabolic fuel and health); accessibility (physical and economic); and availability (the right to nutritious food for all citizens). The sociotype is influenced by multiple factors, including diet–gene interactions, allostasis, microbiota, oxytocin, and culturally through mate selection, family bonds, social communication, political ideologies, and values. Food security, sociotypes, and culture form a complex adaptive system to enable coping with the circumstances of life in health and disease, to achieve sustainable development, and to eradicate hunger. The current geopolitical unrest highlights the absolutely critical role of this system for global security, yet many challenges remain in implementing this paradigm for society. Therefore, sustainable food security must be considered a fundamental human right and responsibility for safeguarding the survival and progress of the sociotypes of humankind (*Homo culturus*) worldwide.

## INTRODUCTION

This review shows how food security and nutrition were the essential factors that enabled communal living in cities and the beginnings of culture and modern civilization. Food, as the essential fuel for the body and mind, mediated this process through the dynamic relationship of individuals with their surrounding social environment throughout life—i.e. their *sociotypes*. The sociotype unites ideas from the sciences and humanities to better understand how humanity copes with life situations in health and disease (see Box 1 for expanded definitions of terms and concepts).

Box 1 **Glossary of Terms and Concepts**

**Complex Adaptive Systems (CAS):** A CAS is a system in which a perfect understanding of the individual parts *does not* automatically convey a perfect understanding of the whole system’s behavior. The system is dynamic and non-linear, with positive and negative feed-back loops and many interconnections.

**Food Systems:** Food systems interact with the environment in multiple ways, as major sources of greenhouse gas emissions and as contributors to water and air pollution, biodiversity loss, deforestation, desertification, and land degradation. Food systems interact with people and society via health (e.g. malnutrition, infectious disease), livelihood (e.g. employment and subsistence), and consumption growth (e.g. driven by diets and population), and are shaped by relationships that may imply power (e.g. gender, wealth, political, and economic relations).

There is a reciprocal relationship between food systems and cultural, social, and ethical concerns, including traditional practices and cultural norms; social identity; animal welfare and rights; religion and spirituality; art; and as part of shared experiences and enjoyment in social life. A sustainable food system is one that is environmentally, economically, and socially sustainable.^1^

**Food Security:** According to the United Nations Committee on World Food Security, food security is defined as meaning that all people, at all times, have physical, social, and economic access to sufficient, safe, culturally acceptable, and nutritious food that meets their food preferences and dietary needs for an active and healthy life.^2^ Food insecurity exists whenever the availability of nutritionally adequate and safe foods, or the ability to acquire nutritious foods in socially acceptable ways, is limited or uncertain.

**Genotype:** Genotype of an organism is defined as the genetic makeup or constitution as determined by the composition of its hereditary material, DNA.

***Homo culturus:*** This review refers to *Homo culturus* to describe the developing cultural attributes of *Homo sapiens* as civilizations evolved.

**Phenotype:** A phenotype is an individual’s *observable traits* whether biological such as height, eye color, and blood type, or behavioral characteristics. The genetic contribution to the phenotype is called the genotype. Some phenotype traits are mainly determined by the genotype, while other traits are largely determined by environmental factors. The environmental factors have been further subdivided into the three dimensions of the sociotype: the individual, relationships, and context. Thus, the phenotype is the product of the interaction of the genotype with the sociotype.

**Sociotype:** The sociotype is an ecological construct that unites concepts from the sciences and the humanities, which, together with the genotype, determines an individual’s phenotype (observable characteristics and behavior) and collectively advances societal culture. The sociotype changes throughout life and with experience. It comprises three domains: the individual (intra-individual), relationships (inter-individual), and context (see text for details).

**Sustainable Diets:** Sustainable diets have a low environmental impact that contributes to food and nutrition security and to the healthy lives of present and future generations. Sustainable diets are protective and respectful of biodiversity and ecosystems, culturally acceptable, accessible, economically fair, and affordable; they are nutritionally adequate, safe, and healthy; while at the same time they optimize natural and human resources.^3^

The importance of food security and nutrition in the development of community, culture, and modern civilization presents an intriguing, evolving story.

The ability to control fire for warmth and protection allowed the early hominids to descend from the trees to lead a nomadic land-based existence and marked the beginning of socialization. For the next thousands of years, the dominant activity of these bands of hunter-gatherers (fewer than 100 persons each) was searching for food. Domestication of livestock and growing crops heralded the Neolithic agricultural revolution some 12,000 years before present (bp). Importantly, food and water security were the preconditions for the survival of the first settlements and the emergence of early societies and cultures.

Societies are made up of the collective outward characteristics and behaviors of their members, known as *phenotypes*. Phenotypes—how we are, who we are, and why we do what we do—are the product of the interactions of an individual’s relatively static *genotype* (hereditary material, genes, DNA) with his/her *sociotype*, i.e. the shifting experiences and environment throughout life. The sociotype comprises three main domains affecting a person’s existence: the *Individual* (intra-personal elements, physical, psychological, spiritual, intelligence, and more); *Relationships* (inter-personal ones, family, friends, social media, etc.), and *Context* (political, health, and educational systems, socio-economic status, cultural values, demographics, media, and more).

The sociotype is expressed through multiple interactive pathways, including diet–gene interactions, epigenetics, allostasis (preparedness for maintaining homeostasis), gut bacteria, oxytocin, and culturally through mate selection, family bonds, social communication, political ideologies, and shared values. While the evolution of sociotypes is measured at the individual level by mate selection and reproduction, at the population level its progress is dependent on adequate nutrition.

Today, approximately half the world suffers from undernutrition and half from overnutrition. Healthy nutrition influences growth and development and susceptibility to disease at all ages, and is a basis for cultural socialization and religious rituals. Nutrition interacts at each dimension of the sociotype to ensure, respectively, the recognized elements of food security: *Utilization* at the Individual domain (essential metabolic fuel of the “highest” octane); *Accessibility* at the Relationships domain (socio-economic influences); and *Availability* at the Context domain (the right of all populations to adequate, nutritious food).

National food security, sociotypes, and culture form complex adaptive systems that determine the direction of human advancement to achieve sustainable development and eradicate hunger. The current geopolitical unrest highlights the absolutely critical role of such systems for global security. However, many challenges remain in implementing this paradigm for society.

## THE BEGINNINGS OF COMMUNITIES: DEPENDENCY ON FOOD SECURITY AND NUTRITION

Humankind has evolved from the higher primates but differs fundamentally in being a cultured/cultural animal.^4^ Prominent descriptors include: larger brain size, communication (verbal and non-verbal), use of tools and technology, abstract reasoning, foresight,^5^ self-awareness, imagination, aesthetics, and the evolution of cooperation.^6^,^7^ Arguably, most of these attributes have only modest origins in the social behavior of animals; rather, they have arisen through the accumulated influence of culture interacting with the potential of the human brain.^8^ The 99% or so overlap between human and chimpanzee DNA indicates that human characteristics arose from qualitative differences that primarily involved gene regulatory mechanisms.^9^

*A three-fold cord is not quickly broken*.

(Ecclesiastes 4:12)

Darwin considered the two formative characteristics for *Homo sapiens* to be the control of fire^10^ and language. The author of the present paper suggests that food security represents a third essential that enabled communal living and cultural evolution. There are many definitions of culture; herein, culture is taken to represent a way of life of a group of people—the cumulative knowledge, behaviors, beliefs, values, and symbols that are passed from one generation to the next by communication and imitation.

After millions of years of evolution, it was the control of fire that enabled the early hominids to descend from the trees. Fire provided protection, light, warmth, sterilization of food as well as its preparation. Sitting together around the fire advanced language development and socialization. Initially, food was roasted. Pottery vessels for cooking were introduced only 40,000 years ago; indeed, the first evidence of extensive dental caries dates to this time.^11^ Activities of daily life centered principally on the search for food, with responsibilities divided between the men, who hunted, and the women, who collected plants and raised their children. These hunter-gatherer groups of fewer than 100 persons existed for hundreds of thousands of years. Then, for unclear reasons—perhaps climatic change at the end of an ice age—the agricultural revolution occurred some 12,000 years bp: people began to settle in one place, domesticated animals, and raised crops. This could only have happened in the presence of food and water security. This in turn led to the birth of cities and culture along the Fertile Crescent and elsewhere,^12^,^13^ concentrating initially on growth, homeostasis, reproduction, conquest, and defense.^14^ The development of pottery for storage, and the wheel, led to commerce between the first urban civilizations.^15^

During evolution, the increase in brain size,^16^ the decreased length of the intestines,^17^ and the limitations of the birth canal^18^ together led to three developments necessary for a child to become physically independent, and later, socially mature—postnatal brain maturation, language acquisition,^19^ and prolonged parenting.^20^

**Figure 1:**
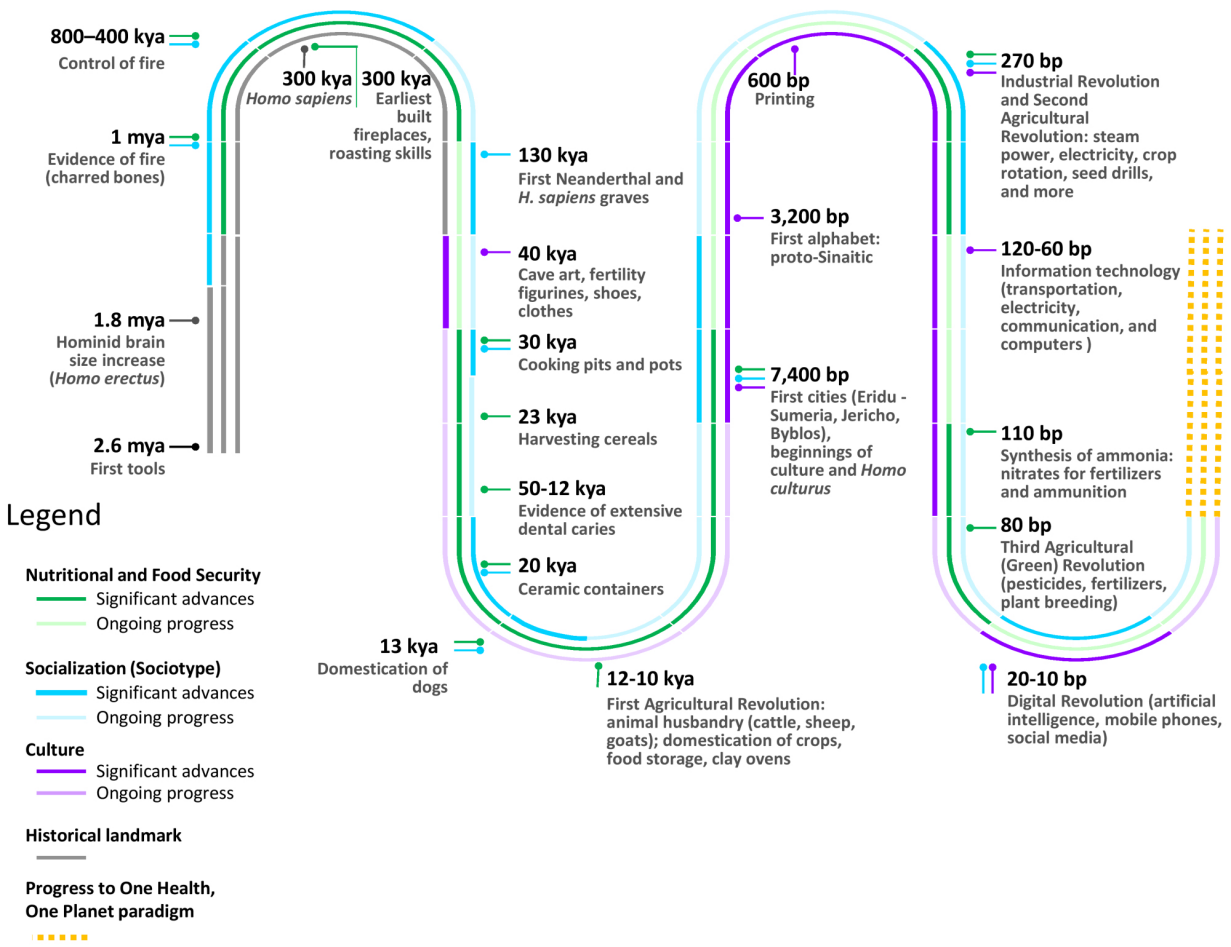
Progress from Food Insecurity to Food Security: Development of Socialization and Culture. All dates are approximate. The dotted orange lines at the end represent future progression to the One Health, One Planet paradigm. bp, before present, kya, thousand years ago, mya, million years ago.

Figure 1 presents a summarized time line of human development from the perspective of fire control, food, and nutrition to show how they were indeed antecedents to culture and its rapid development to the present day. Taken together, the major impact has been the increased availability of knowledge—the substrate of cultural evolution.^21^ In this connection, Hans Jonas noted that the three descriptors of *Homo sapiens—*tools, images, and graves—represent the beginnings of physics, art, and metaphysics, respectively.^22^

## INTRODUCING THE SOCIOTYPE

**Figure 2:**
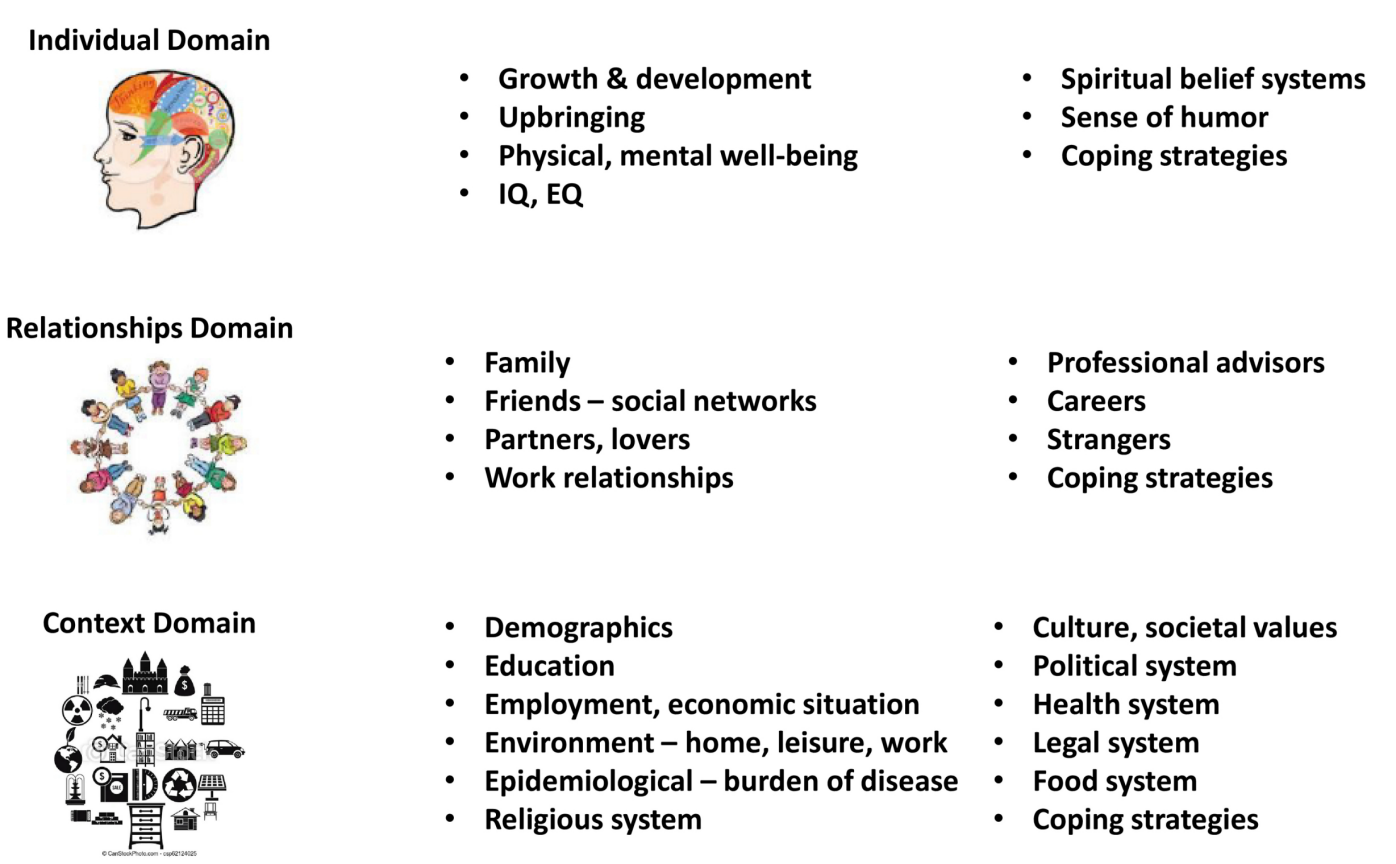
Infographic of Some Influencers of the Three Sociotype Domains. The three sociotype domains are the *Individual*, *Relationships*, and *Context*. The items listed on the right are some of the influencers on each domain. Sociotype awareness in each domain helps the individual cope with life’s circumstances in health and disease. It should be noted that some stressors may affect all three domains, such as diabesity, food insecurity, and most recently, COVID-19. Modified from Peng and Berry (CC BY 3.0).^27^ EQ, emotional intelligence; IQ, intelligence.

Human cultural evolution is characterized by its sophisticated group behavior.^23^ Cultural maturity requires, in addition to individuation and separation, interpersonal interactions with family, peer groups, and society. Hence, this author originally proposed the term “sociotype”^24^,^25^ as an extension of Engel’s bio-psycho-social model^26^ (Figure 2). The s*ociotype* is an ecological construct that combines concepts from the sciences and humanities and interacts with the genotype to determine an individual’s phenotype—how you are, who you are, and why you do what you do. Collectively, they define the evolution of societal values and culture. The sociotype has three domains, the Individual (intra-personal), Relationships (inter-personal), and Context, and influences the phenotype developmentally, behaviorally, and socially through these domains. Examples at the Individual domain include influencing nutrition, imprinting,^28^ parenting, and personality development^29^,^30^; at the Relationships domain examples include shaping family, peer group, social, and work interactions^31^; and at the Context domain, responses to education, the prevailing culture(s), political system(s), socio-economic status, and the demographic environment. The inputs of the genotype on the phenotype are relatively fixed, apart from variable epigenetic, gene regulatory effects, and the impact of mate selection on future generations, while the sociotype inputs change continuously with age and accumulated life experiences. The sociotype concept provides a framework for understanding more fully what constitutes the “environment” when considering gene–environment interactions. Figure 2 presents an infographic showing some of the many factors within the sociotype domains that affect coping strategies for life circumstances,^32^ such as diabesity,^25^ food insecurity,^33^ and, most recently, COVID-19.^27^ Life stresses may affect and overlap with more than one domain: the diagnosis of diabetes will first affect the individual and then relationships, if (say) the disease leads to impotence. Balancing time and energy between leisure and work, or home and office, affects both the relationships and context domains; food insecurity and economic crises can affect all three.

Box 2 lists some of the kinds of questions that the sociotype deals with, reflecting its scope and flexibility in addressing various life situations, nutrition, and some of the anomalies in societal values and cultural assumptions.

Box 2 **Some Questions Relevant to the Three Sociotype Domains Addressing Different Life Situations, Nutrition, and Anomalies in Cultural Values and Assumptions**

Questions Asked at the *Individual* Domain

- What will be the effects of different parenting models (currently at least six based on gender and adoption models) and preferred pronouns on your child’s upbringing and later life adjustments?
- Is the sensation of hunger in your head or your stomach?
- Is obesity a form of food waste?
- How would your life have been different if you were 10 cm taller or shorter, or 15 kg heavier or lighter?
- How do you live with physical, emotional, or cognitive special needs?
- How do you cope with a severe physical condition, e.g. chronic disease such as diabetes, stroke, cancer, or arthritis, post-transplantation, after a severe accident, or any other?
- Why are packages so hard to open nowadays—or is it a sign of aging?

Questions Asked at the *Relationships* Domain

- At what age do children cease to seek parental approval?
- What determines the choice of a spouse or partner?
- Why do advertisements show pictures of scantily clad men and women to sell perfume, cars, potato chips, and beer?
- What determines the dynamics of relationship in sibling rivalry (because no two children are born into the same family)?
- Why do children think they know better than their parents—in every generation?
- How do families cope with a child suffering from epilepsy, diabetes, or other serious health or disability problems?
- How do families deal with family gatherings, divorce, bereavement, or other high-stress situations?
- Who is responsible for looking after elderly parents or grandparents?
- How do families care for a parent/spouse with dementia?
- How do families cope with food insecurity?

Questions Asked at the *Context* Domain

- How do you deal with job dismissal, retirement, or moving from home?
- How do you deal with economic crises, natural and manmade disasters, or wars?
- Why are there separate chess championships for men and women?
- Why do drivers and pedestrians fail to see the other’s point of view, when only a short time earlier (in the car park) their perspectives were reversed?
- Why are dog owners, but not horse owners, expected to clean up after their animals?
- How do you handle aging within the social context (retirement, senior benefits, nursing homes, loss of independence, etc.)?
- How would geopolitics be altered if Middle Eastern oil sources had been distributed differently?
- Why do Westerners and Asians have difficulty identifying each other’s faces?

The term sociotype has also been used in a more limited sense, to describe only the social environment (i.e. relationships) and its relevance to cultural evolution.^34^,^35^ However, herein a more holistic and ecological framework is developed to include the individual and context domains. In addition, the possible cultural and biological pathways through which the sociotype determines the phenotypes of individuals and society are discussed. These include mate selection, social media, and political systems, on the one hand, and, on the other, diet–gene interactions, epigenetics, allostasis, microbiota, and more. Furthermore, the critical importance of food security and nutrition throughout the life cycle is emphasized. Regarding evolutionary progress, at the individual level mate selection and reproduction are the main yardsticks, while at the population level it is adequate nutrition.

The study of the sociotype involves many disciplines, including nutrition, biology, psychology, anthropology, ethology, medicine, sociology, economics, political science, and the environment. As opposed to natural processes, over which people have no control, social behavior and technology have enabled people to influence their environmental circumstances, whether through urbanization, transportation, air pollution, or birth control. The study of “collective behavior” supports the concept of societal sociotypes—the rules whereby individuals interact—which results in transitions of “group-level” phenotypes or cohesive behaviors. These, in turn, provide novel selection pressures and new sources of “knowledge,” including opportunities to profit from the experience of others. Such models have been applied to insects, fish, birds, road traffic, crowds, and the stock market.^36^

It should be noted that there is no definition of a *normal* sociotype; rather, the nature of any society represents the sum of its interactions with individual sociotypes that are produced and result from the individual’s particular culture and life experiences.^37^,^38^

## FOOD SECURITY, SOCIOTYPES, AND CULTURE AS A COMPLEX ADAPTIVE SYSTEM

**Figure 3:**
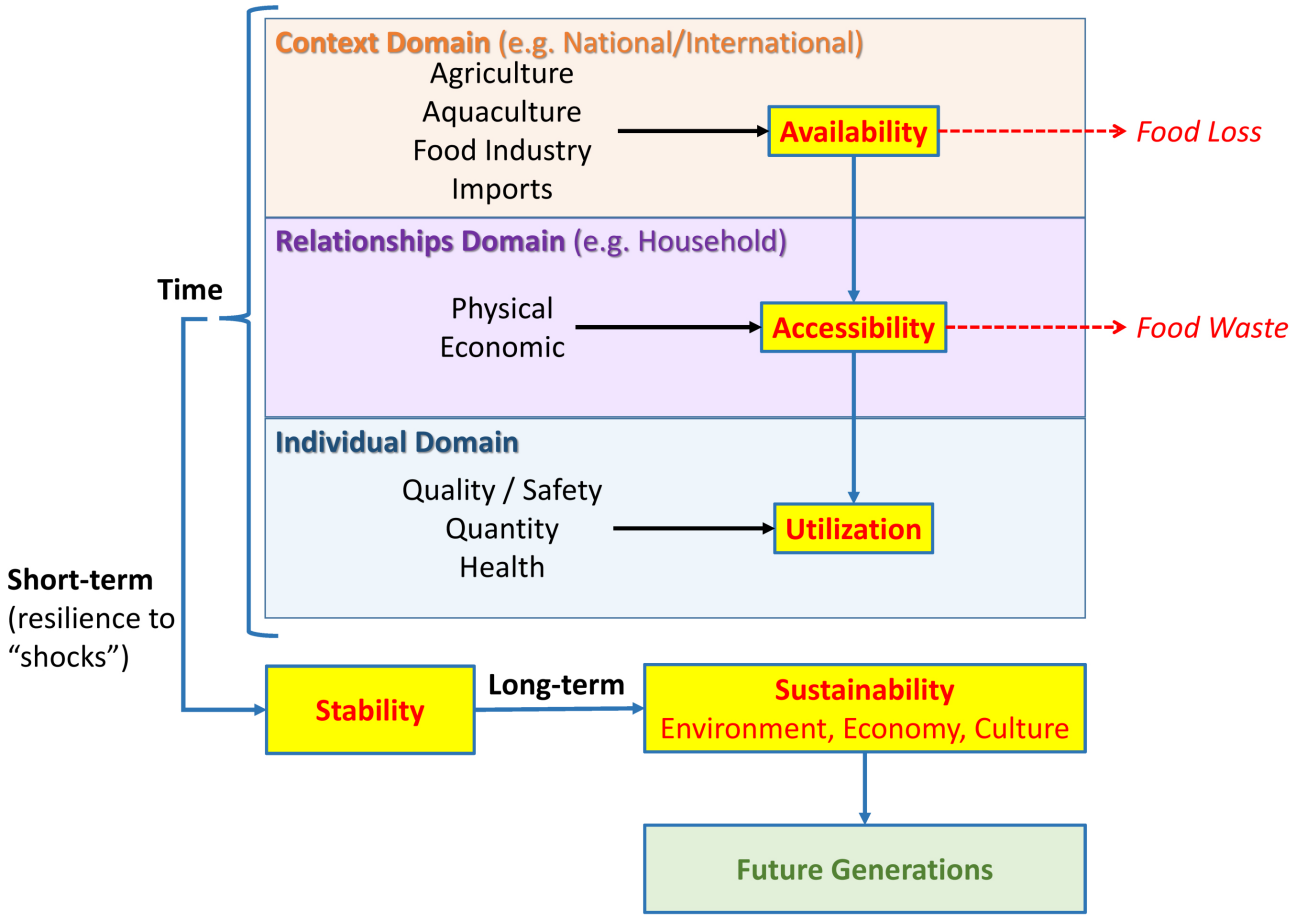
The Food Security Pathway in Relation to the Sociotype Domains. The original three dimensions of food security were *Availability*, *Accessibility*, and *Utilization*. These correspond respectively to the Context, Relationships, and Individual domains of the Sociotype. The food security pathway is represented by the blue arrows. Note that approximately one-third of food overall is lost and wasted along the pathway (dotted red arrows). Stability and Sustainability represent the time dimensions of food security at the short- and long-term levels, respectively. Short-term stability rests on the ability to be resilient to shocks, whether they be economic, or manmade or natural disasters. The relative importance (weightings) of the food security dimensions (yellow boxes) in a given country depends on its food chain and system. Sustainability refers to the long-term goal of ongoing food stability that takes into consideration environmental, economic, and cultural aspects without jeopardizing future generations.

Surprisingly, food security and nutrition seem to have been ignored as the driving forces permitting cultural evolution. The major advances in civilization could not have occurred without a regular food supply.^39^ In describing food security, the United Nations Committee on World Food Security point out that it is present only when all people, at all times, have, in a culturally acceptable manner, physical, social, and economic access to sufficient, safe, and nutritious food that meets their food preferences and dietary needs for an active and healthy life.^2^ The dimensions of food security have evolved over recent decades (Figure 3). Initially they focused on food availability and its production; then they were expanded to include the household physical, economic, and sociocultural accessibility to food, and food utilization by the individual, corresponding to the sociotype Context, Relationships, and Individual domains, respectively. Since the food crisis of 2008, stability was added as a fourth dimension of food security to cope with short-term disasters whether financial, natural, or manmade. Unfortunately, the current geopolitical crisis in Ukraine is evolving to produce food, commodity, and energy crises with horrendous ramifications that may well force people in many parts of the world to choose between freezing or starving (heating or eating).

Nutrition interacts at all stages of the life cycle to ensure growth and development in health and disease as part of evolving sustainable food systems^40^,^41^ and planetary sustainability.^42^ We recently introduced sustainability as the long-term (time) fifth dimension for food security,^43^ to ensure the right of future generations to healthy food produced through ecologically sustainable methods. The Mediterranean diet is a case-study for sustainable diets combining health, socio-cultural, economic, and ecological benefits.^44^

**Figure 4:**
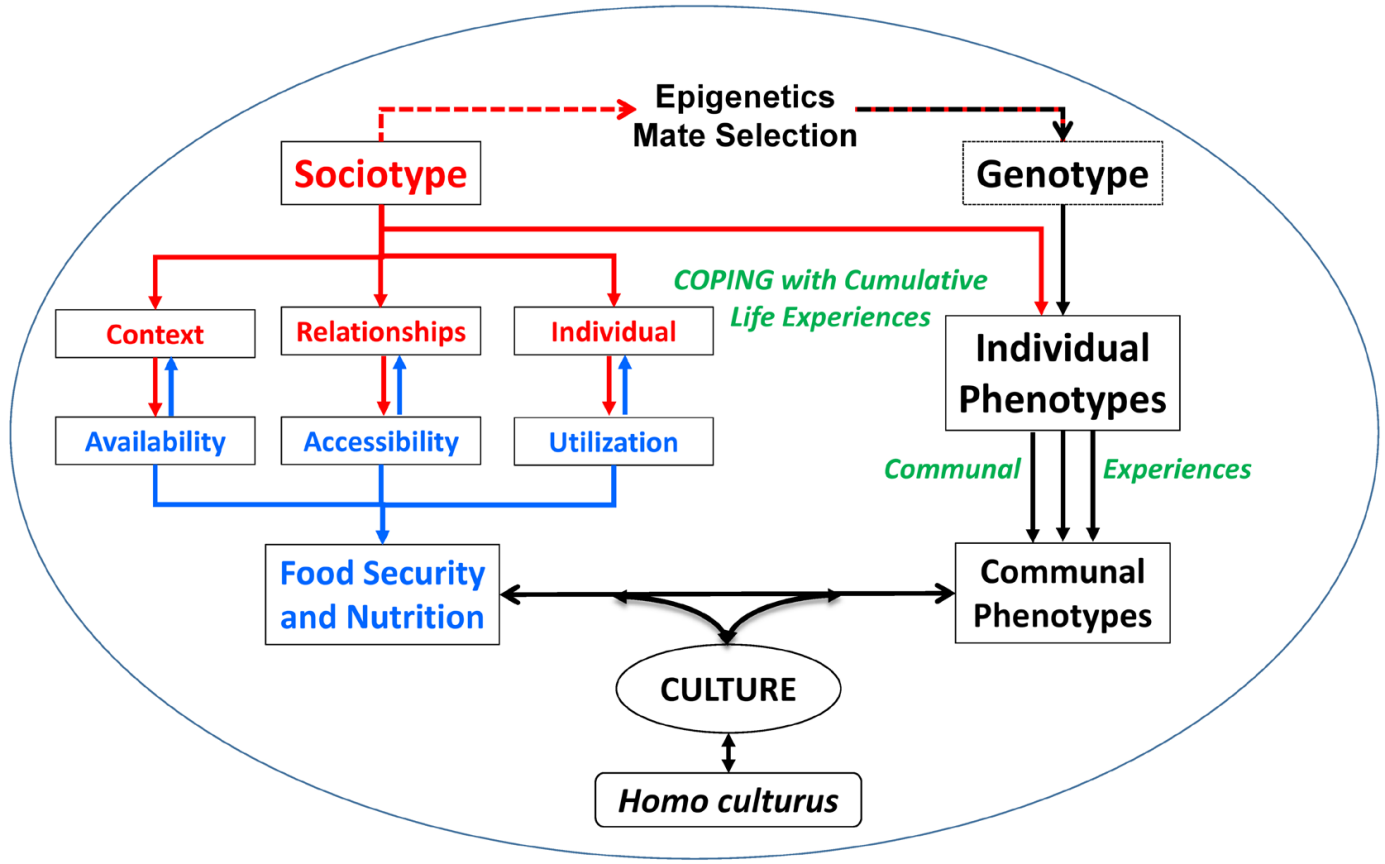
The Complex Adaptive System Involving Food Security and Individual and Communal Phenotypes Which Together Enabled the Development of Culture as Represented by *Homo culturus*. The sociotype interacts with the genotype to produce the individual phenotype. The inputs from the sociotype (red) vary continuously by coping with cumulative life experiences (green), while that of the genotype is relatively fixed apart from variable epigenetic, gene regulatory effects and the impact of mate selection on future generations. Collective phenotypes and their experiences make up the community that is maintained by food security (blue), the dimensions of which interact with the three sociotype domains. The experiences of the individual phenotypes, as a whole (communal experiences), lead to the development of communal phenotypes, which are affected by and can affect food security and nutrition. Culture develops, and *Homo culturus* is sustained through the (healthy) feedback between communal phenotypes and food security and nutrition. The encompassing circle (blue border) represents the complex adaptive system required to sustain and analyze the actions and behaviors of *Homo culturus*.

The sociotype, together with food security and culture, forms a complex adaptive system (CAS) (Figure 4).^24^,^45^ A CAS uses concepts from systems theory, population ecology, and information processing. It is characterized by complex behaviors (such as culture) that often result from non-linear temporal interactions among many component systems, at different levels of organization, involving interdependency, and producing dynamic outcomes in unpredictable ways. Only some of the interrelationships are shown in Figure 4, since it would be too complicated to show every item connected to the others, which is what actually happens in a CAS with many positive and negative feedback loops. In this paradigm, humankind is part of complex natural systems which affect profoundly our planet and its biosphere. Some practical examples of such interactions are: poverty in social and economic systems; food insecurity in agricultural and economic systems; and compromised immunity in human biological systems.^45^

The CAS involving sociotypes, food security and nutrition, and culture evolved to advance *Homo culturus* (referred to in a descriptive, developmental sense rather than to designate a biologically different species; *Homo culturus* incorporates various characteristics such as *H. ludens*^46^ or *H. economicus*,^47^ and the long-lived *H. religiosus*/*deus*). Nutrition is to the body what education is to the brain, and culture to society. There are multiple sociotype pathways that reciprocally link to food security, for example the reactions to global warming or COVID-19.^27^ These responses, initially in the Context domain, affect not only the food supply chain and prices, but also modify idea systems (culture) as new language (epidemiology), concepts, and even new fears (Kafkaesque) enter the vocabulary of everyday life,^48^ affecting all levels of society.

### Interactions between the Sociotype and the Genotype

Within the CAS, there are two evolutionary mechanisms for *Homo culturus*, genetic and socio-cultural. Both involve transfer of “information”—DNA in the genotype, and, in the sociotype, information stored and transmitted across generations whether from internal processing (ideas, creativity, traditions), or from external sources such as books or information technology (e.g. the internet).

Variations at the genotype level are provided by recombination and mutations. Sociotypic influences on epigenesis and mate selection can affect gene expression in the individual and the genetic makeup of future generations, although there is no evidence for germ line involvement.^49^,^50^

Mate selection is a prime example of the sociotype in action; it is the non-random aspect of reproduction in natural selection.^51^,^52^ What does he see in her (*anima*) or she in him (*animus*)?^53^ This determines the choice, which involves many biological, psychological, and cultural factors.^51^ While animals may choose major histocompatibility complex (MHC)-incompatible mates, there is no evidence for this in our species.^54^ It is hoped that the magic and mystery of courtship will always retain something of the unfathomable, whether attributed to “opposites attract” or “like likes like”—the latter apparently the dominant paradigm in human populations.^55^ There is little doubt that courtship and family life is centered on cooking, eating, and meal times.^56^ It is not known how demographic transition (a drop in death rates in industrialized societies followed by a decrease in fertility),^57^ or the fact that birth rates in some countries may be lower in the higher social classes, will affect the transmission of cultural changes or the future intelligence of societies.

### Nutrition During the Life Cycle and Effects on the Sociotype

The interactions between nutrition and the sociotype domains must also be considered. Table 1 shows some of major influences of nutrition during the entire life cycle in health and disease, and possible mechanisms of action.

**Table 1:** Some of the Mechanisms by Which Nutrition and Sociotypic Influences Affect Development in Health and Disease Throughout the Life Cycle.

Development Period	Influences	Mechanisms / Determinants / Outcomes
Pre-pregnancy	Neural tube defects	Folic acid^58^

Pregnancy	Intra-uterine environment	Metabolic programming,^59^,^60^ epigenetics,^61^ stress telomere length^62^

Neonate	Bonding	Breast feeding,^29^ microbiome^63^

Childhood	Growth, development	Redox homeostasis^64^,^65^; balanced nutrition; food quality, quantity, and safety; lifestyle^66^

Adolescence	Menarche, fertility, food, mood	Body fat^67^; food emotion^68^; neurotransmitter synthesis dependent on essential amino and fatty acids, vitamins^69^
	Social interactions	Oxytocin^70^
	Eating disorders	Epigenetics^71^
	Body image	Cultural norms, social media

Adulthood	Mate selection	Pheromones*,^72^ cultural taboos such as Kibbutz intermarriage “taboos”,^73^ synchronization of menstrual cycles,^74^ handicap principle^75^
	NCDs: Obesity, cardiovascular disease, diabetes, cancer	Stress—allostasis,^70^,^76^ circadian rhythms,^77^ cultural norms and lifestyle, microbiome,^63^ diet–gene–enzyme interactions,^78^ diet heart,^79^ epigenetics,^80^ immune function^9^

Old age	Longevity	Caloric restriction*^81^

* Controversial.

NCDs, non-communicable diseases.

#### Sociotype Individual Domain

The vast literature on fetal origins of disease is beyond the scope of this review, but it emphasizes the critical role of nutrition (from nine months before conception) in modifying pregnancy, development, and lifetime morbidity.^59^ Studies from the Second World War (the Dutch Famine and the Siege of Leningrad) and after the Great Leap Forward in China (1958–62) have shown consistent associations between malnutrition during pregnancy and adult body size, metabolic syndrome, and schizophrenia, which may be explained by epigenetic mechanisms.^49^,^82^

Evidence from twin studies suggests that the genetic contribution to the microbiome in humans is slight. Diet is not only dominant over genetics in affecting the microbiome composition, it is also superior in predicting multiple host traits, such as blood glucose levels and obesity, raising the possibility of personalized nutrition.^63^,^78^

#### Sociotype Relationships Domain

Breast feeding and infantile nutrition have provided fertile ground for developmental theories of family dynamics,^83^ human growth and drives,^84^,^85^ and object relations,^86^ postulating that the earliest influences are the longest and the strongest. Oxytocin is considered the social hormone,^70^ and stimulation of its pathway may alleviate symptoms in an animal model of autism.^87^ It is not yet known what happens to oxytocin levels and expression in response to the social isolation caused by the lockdowns and quarantines during the current COVID-19 pandemic.

#### Sociotype Context Domain and Food in Culture

Eating, drinking, breathing, the senses, and trauma guide our interactions with the physical environment. Of these, the need for food and water has been crucial for establishing centers of civilization. The definition of cuisine is a food specifically cooked based on a culture’s ingredients, region, and traditions; there are hundreds of known cuisines worldwide.^88^ (As a humorous aside, it has been noted that there is neither a specific British cuisine nor a custom to say the equivalent of “Bon appétit.” This is remarkable since an appetite is definitely needed to eat the meal, and accounts for the surfeit of ethnic restaurants found in the UK, where the most popular dish eaten outside the home is apparently chicken Tandoori.)

Albala has edited a very informative volume on food culture covering a number of topics including feminist food studies, food and communication, and food in the arts.^89^ From an evolutionary perspective human diets differ from animals’ by nature of (to name a few) the different cuisines, food distribution, relationship exchange while eating, food preferences, religious prohibitions, and ritual symbolism surrounding food.^48^,^56^,^90^ A distinction is made between the *anthropology of food*, which deals with eating and drinking in connection with other aspects of social life, and *nutritional anthropology*, which considers food-related social meanings and beliefs affecting well-being at all levels of the sociotype.^91^,^92^

Food taboos are culture-dependent when considering countries where people will or will not eat blood, cats, dogs, horses, insects, snakes, frogs, and snails, including types of ritual slaughter—kosher or halal.^48^,^90^ In parts of Africa, colostrum is considered to be poisonous, thereby depriving neonates of antibody protection. Pregnant women will not eat chicken necks, which are the preferred food for the elderly. In Ethiopia, only the poor eat liver, and no one in Ghana will deliberately lose weight, it being a sign of disease or infertility; in fact, if a wife does not gain weight after marriage, her husband is accused of neglect. Regarding etiquette, fingers were used long before forks, which only became common in Europe after the eighteenth century, and today, in the appropriate culture, belching may still indicate positive feelings of satiety and appreciation of the meal.

There are many examples of eating and food in art, from the forbidden fruit in the Garden of Eden to the Last Supper.^93^ Some examples in literature relating to hunger are found in Dante—Count Ugolino locked in the tower with his children (Canto Inferno XXXIII, and the wonderful statue by Rodin); in Kafka (“A Hunger Artist” and “Investigations of a Dog”), while he himself died of inanition (due to tuberculosis of the larynx); and the pre-existentialist novel “Hunger” by Knut Hamsun. At the other extreme, obesity is dealt with humorously in “The Three Fat Women of Antibes” by Somerset Maugham and in “A Piece of Pie” by Damon Runyon describing the incredible eating competition. Lévi-Strauss affirmed that “the musical creator is a being comparable to the gods, and music itself [is] the supreme mystery of the science of man” 94(p; indeed, music has been found to affect appetite as part of the socializing importance of meals.^95^

The reciprocal effects between food and modern culture were summarized by Parasecoli: “The presence of food in everyday life is pervasive, permeating popular culture as a relevant marker of power, cultural capital, class, gender, ethnicity, and religion … Meanwhile, our *own flesh becomes fuel* [italics added] for all kinds of cultural battles among different visions of personhood, family, society, polity, and economics.”^96^(p These scenarios are still evolving.

Other topics for future discussions include genetically modified crops, food justice, food sovereignty, and food ethics for producers and consumers, regulation of junk food advertisements to children, animal rights, agro-tourism, and the economics of food production.

### Disorders of the Sociotype, Food Security, and Culture

Disorders of the sociotype may involve the individual, family, or society. Disorganization at any level of human development and interpersonal interactions may lead to a maladapted individual, and to physical and mental illness.^97^ Eating disorders are an example of a culture-bound disease influenced variously by family, the media’s perception of desirable bodies, and ideals among adolescents, especially in relation to body dysmorphism and pornography. The problem is not helped by remarks such as “you can never be too rich or too thin” (Duchess of Windsor), or “nothing tastes as good as being thin feels” (Elizabeth Berg), together with a tendency to mortification.^68^,^71^,^98^,^99^ Cultural values also influence the acceptability of obesity which, in some societies (e.g. in Africa and the Middle East), is encouraged in men for status, and in women for fertility, and was idealized by the Venus of Willendorf, which dates from about 27,000 years bp. The ever-constant pain of hunger was felt in the concentration camps of the Second World War^100^ but did not deter the remarkable sense of duty of the Warsaw Ghetto doctors who documented Hunger Disease for the first time.^101^

The current obesity pandemic^102^ is surely due to the toxic obesogenic environment affecting both sides of the energy-balance equation by encouraging magnum portion sizes and labor-saving devices. Genes have not changed over this time period. Instead, human physiology (eat to live) has been overridden by psychology (live to eat). The increasing prevalence of convenience (ultra-processed) food reduces the need for, and ability of, people to cook fresh meals and aggravates obesity at the population level.

Today, almost equal numbers of people suffer from obesity as from undernutrition (2 billion), despite there being enough food available to feed everyone. The fact that one-third of food is wasted from farm to fork is a global indictment in the fight against hunger. The Green Revolution, which did initially improve crop yields, was achieved at the cost of an increased use of pesticides and fertilizers, globalizing agriculture, and displacing small farmers and the consequential loss of traditional practices and biodiversity.^103^ This is the tragic triumph of Big-Agro economic interests over small farmers, social justice and equity, and a sad commentary on national and international responsibilities and values.^104^

Humans eat food and also excrete, which adds to problems of disposal and hygiene since many people lack toilet facilities and potable water. Less than 25% of the world’s population (the Global elite) have clean water, food security, own or rent houses, have a cell phone and internet access, and can obtain tertiary education.

At the level of society, are there “dyscultural” conditions akin to dysgenics? Group or herd behavior (sports supporters), political demonstrations between the Right and Left, mass hysteria, cults following “isms” (messianism, communism, fascism, religious fanaticism, etc.), and riots and mob violence may represent such phenomena.^105^,^106^ Society is poorly equipped to combat the challenges presented by anti-vaxxers and promoters of fake news. Will globalization eradicate many cultures by promoting uniformity? Is the internet a double-edged sword that provides unlimited sources of information, yet also disseminates unfiltered disinformation about, *inter alia*, food, relationships, and culture? Social inequalities are the major determinants of disease today, with life expectancies varying by more than 30 years among countries.^107^ The effects of social inequality are probably as great, if not greater, than the biological ones, and the sociotype framework can help define them. Answers will come from interdisciplinary studies of societal behavior, as well summarized by Winterhalder and Smith.^108^

### Methodological Considerations for Assessing Interactions between Sociotypes, Food Systems, and Culture

Much work is necessary to describe the biological and gender-specific pathways of the sociotype relating to stress, disease, and coping, such as allostasis^70^,^76^ and epigenetics.^109^ The sociotypic domain classification is perhaps more helpful in framing research questions^32^ than the micro-, meso-, macro-, and exo-classifications used in other ecological models.^31^ A dedicated questionnaire has been developed for relationships.^34^ However, interactions of sociotypes, sustainable food systems, and cultural organizations require more multi-level analyses for CAS.^110^ A combination of applied, theoretical, and experimental methods (e.g. mathematics and computer simulation, mixed methods) is required, since these systems are closely linked, to make sustainable food ecosystems.^41^ An excellent example for such an analysis is the redox system,^65^ which includes chloroplasts—the sites for photosynthesis—the indispensable process for harnessing solar energy to initiate the food chain essential for all hominid evolution. Stuart Kauffman introduced the concept of fitness landscapes for these situations.^111^ Future challenges are to understand and develop the sociotype for coping strategies throughout life, and to teach and research CAS to advance for all societies the One Health, One Planet paradigm, an integrated, unifying approach that aims to balance sustainably, and optimize, the health of people, animals, and ecosystems.^40^

## *HOMO CULTURUS* AND CULTURAL SUCCESS

Gould noted that human cultural evolution differed markedly from biological evolution, in that it is Lamarckian in character (in its inheritance of acquired features). In other words, what one generation learns is passed on directly by teaching and writing to the next.^112^ Huxley defined cultural evolution as “psycho-social selection,”^113^(p and claimed that it was ignored by Darwin, even though it may follow Darwinian principles.^114^ The details of such processes (variation, selection, and inheritance), however, remain unclear, although various candidates have been suggested such as memes115 or culturgens.^38^ Genetic inheritance is essentially vertical, whereas culture has, in addition to transgenerational effects, major horizontal elements. The speed of cultural change is far greater than that of genetic selection, as cultural invention and sociotype–phenotype interactions replace genetic change as the effective source of variation.^116^ Prenatal diagnosis and genetic engineering have extended control over future generations, as artificial selection replaces natural selection in directing genetic evolution.

Examples of culture–gene interactions are well known. Persistence of the hemoglobin S gene may be attributed to heterozygote resistance to malaria.^9^ Similarly, the heterozygous state of the *apolipoprotein L1* (APOL1) gene associated with chronic kidney disease may protect against *Trypanosoma brucei rhodesiense* infection.^117^ In the same way, the selective pressure of shared cultural traits—animal domestication and adult milk consumption—has allowed the persistence of the lactase enzyme.^118^ Other examples include amylase 1 gene duplication for improved starch digestion, fatty acid dehydrogenases, alcohol intolerance, folate, and vitamin D requirements.^9^ Also of note are the changes in the aryl hydrocarbon receptor to detoxify potentially carcinogenic heterocyclic amines from smoke inhalation, which was especially relevant to cave dwellers.^119^

There are interactions between the sociotype domains and culture as typified by Tolstoy’s intriguing discussion in the epilogue to *War and Peace*, on the march of history—whether due to great leaders (Individual), interactions of peoples (Relationships), or new ideas (Context).

Over the past 500 years, major changes in living habits, science and medicine, politics, fashion,^120^ and education have influenced institutions, values, demography, and longevity—all without making any detectable changes in the human gene pool. Women’s fashions constantly evolve, as demonstrated by elevator-like changes to skirt lengths, while men have still not decided how many buttons are needed for jackets. Cultural extinction has occurred, as exemplified by the demise of men’s hats and spittoons in the last century. The passing of spittoons followed a public health campaign, as an excellent example of an interactive sociotype model. The art of letter writing is quickly lapsing in both the older and younger generations due to Twitter, instant messaging, e-mails, and, most recently, TikTok. Digital photography and selfies have replaced 35 mm film reels, obviating the need to compose and restrict the number of photos taken. On the other hand, techno-cultural evolution has led to tremendous advances in transportation and communication, bringing citizens of the world closer together and hastening globalization for better or for worse.

In 1968, Lévi-Strauss looked for group cultural universals such as religion, marriage laws, and rituals around death.^121^ Religiosity may be related to the genes proposed for spirituality to explain the almost universal development of religions in all world cultures,^122^ probably the result of existential angst of the unknown and uncontrollable. There is evidence that Neanderthals and *Homo sapiens* (who coexisted 150,000–30,000 years bp) were the first to practice deliberate burial of the dead, with some evidence for ritual activities,^123^ and the Egyptians added food at burials to sustain the dead in the after-life. The practice of male circumcision in different societies is an example of convergent cultural evolution whether for religious purposes, as a rite of passage, or to protect against sexually transmitted diseases.

If differential reproduction is the mark of success in genetic evolution, what is the analogous criterion in cultural evolution? In the family, there is usually a Marxist sharing of resources, whereas capitalism and competition are the dominant paradigms outside of it. Here the survival of the fittest refers to material success. This may be an over-simplification since we live in a mixed complex society of both capitalists and socialists: how we make products is competitive—how we organize our work, government, and communities is more socialist. Overall, we should hope to aspire to social-democratic mixed economic societies.

Culture could be assessed by books per household or general knowledge (intelligence quotient plus emotional intelligence), scientific productivity, or a harmonious society where minorities’ and women’s rights are respected. From the psychological (a lone human attribute) view, cultural success may be related to pleasure, profit,^124^ power, status, and fame—which also includes creativity,^125^ self-actualization,^126^ and today, sadly, the number of “likes” on social media.

Cultural success is different for the individual and the group. It is culture that has separated reproduction from sex, introducing non-biological categories such as virgins and celibates. There are other fields, such as creativity in art, literature, and music, that cannot be said to progress, but only change, unlike advances in science and technology. For example, is urban wall graffiti a type of art? Francis Schaeffer noted that there is no such thing as “bad” art since all art is a reflection of society; hence, if one perceives art as “bad,” this is more a critique of the societal environment of the artist than of the art itself.^127^

What does body tattooing say about form, anatomy, and gender issues? Will heavy metal be played 200 years hence? Such cultural tests of time are equally appropriate for literature, art, and music.

Some see the modern trends of individualism, instant gratification, and the diversions of *panem et circenses* (Juvenal, 60–130 Common Era) reappear as *pizza and football* together with anti-convivial fast food consumption. This leads to another important psychological attribute of humankind: a sense of humor. We have even been defined as the laughing animal.^128^ Most children play and laugh at the same things, but such similarities and cooperation unfortunately decline with adulthood. Humor is also important as a coping mechanism as in obesity, “not to take heavy matters too seriously.”^129^ As Horace Walpole remarked: “this world is a comedy to those that think, a tragedy to those that feel”—a solution to why Democritus laughed and Heraclitus wept.^130^

## EVOLUTION OF ETHICS AND THE FUTURE NOURISHMENT OF THE WORLD

Socio-cultural evolution has progressed unevenly. During the nineteenth century, the value of human life increased as capital punishment was restricted and slavery abolished. Yet, women’s suffrage and abortion reached Switzerland and Italy, respectively, only in the late twentieth century. Genocide, either physical (as in Rwanda and Srebrenica), cultural (as in Tibet), or both (Uyghurs), continues to occur long after the Holocaust. Political unrest, tribalism, and lack of unity in sub-Saharan Africa are on-going tragedies, as is the plight of refugees worldwide. Religious fundamentalism, child labor, and human trafficking remain as major, current socio-cultural and political concerns. An interesting thought experiment for sociotypic evolution is to consider how society would look if everything remained static, except that women were physically stronger than men. Would there be more or fewer wars? Would there be more or fewer births?

Dunning remarked: “In the moral order of things, [people] rank somewhere between angel and animal”^131^(p; so it is a moot point whether ethics and morals have evolved at the same rate as science and technology. The emotionally charged interpersonal relationships described in the Bible and in the Orestes trilogy, involving passions, jealousy, vengeance, betrayal, corruption, supernatural beings, and more, are still very much alive today.

Julian S. Huxley, who foresaw many of the ideas developed herein, noted: “The enjoyment of beauty and interest, the achievement of goodness and efficiency, the enhancement of life and its variety—these are the harvest which our human uniqueness should be called upon to yield.”^128^(p But, however unique, humanity cannot do it alone. Instead of relying on the “blind” forces of natural selection, the challenge for humankind is to shape its evolution through the action of its sociotypes to preserve society so that the lessons of Easter Island are not repeated.^132^ What were the Islanders thinking when they cut down the last fruit tree? And in the not-too-far-distant future, will we even notice when the last glacier melts?

A regular source of water and nutritious food is the *sine qua non* for communal living, the *fons et origo* of culture. Food is required as a continuous metabolic input, critically affecting growth and development throughout life through the sociotypic influences on health and disease.^25^ These are encapsulated by observations from its domains. First, the Individual domain uses the words of Brillat-Savarin (1755–1826)^133^: “Tell me what you eat and I will tell you what you are.” This celebrated saying can be extended to the Relationships domain as *Tell me how a family eats, and I will tell you how it functions*. The importance of social meals, such as Thanksgiving, Christmas, Eid al-Fitr, and Passover Seder gatherings, is common to many different cultures, demonstrating the common adage that *the family who eats together, stays together*. And, finally, in relation to the Context domain: *Tell me how a nation eats, and I will tell you its values*. There must be a realization that food-insecure people live within every country and always will. Adequate nutrition should be a priority and responsibility for every nation, equal to that for health, education, and defense.^104^ The interactions between the sociotype and culture are essential for understanding the “other” in health and disease.^134^

Ensuring global food security and cooperation between sociotypes will enable *Homo culturus* to achieve the goals of social justice and sustainability, thereby guaranteeing the physical and economic wellbeing of societies—a food-secure nation is a healthy, resilient, and productive nation.^135^ Sustainable food security, therefore, must be judged a fundamental human right and responsibility to safeguard the survival and progress of *Homo culturus* in all parts of the world.

## CONCLUSIONS

*Man does not live by bread alone …*

(Deuteronomy 8:2–3; Matthew 4:4)

*Homo sapiens* is a continual metabolizer but an intermittent feeder, and early hominids spent most of their time trying to find food. Darwin considered their two formative characteristics to be language and the control of fire. Herein, food security is suggested as the third essential in allowing hunter-gatherers to settle in large groups to develop their individual and collective sociotypes for coping better with life circumstances and to evolve societal culture.

The benefits of fire ultimately led to language and socialization. These, in turn, led to relationships and mate selection as the determinants of the next generations. The agricultural revolution enabled hunter-gatherers to switch to urban dwelling and more stable crop-based societies. The lack of food security prevented living permanently in a single place. This situation changed due to a number of advances: animal husbandry; domestication of crops and fruit trees; and storage vessels necessary for trade, and food security. For the first time, people felt food-secure and were able to develop skills and cultures that extended far beyond the basic need for food provision.

However, there is no anthropological consensus that improvement in food security was always guaranteed. Armelagos and Cohen^136^ showed declines in health and nutrition at the dawn of agriculture in many parts of the world. Food shortages are frequent in farming societies. Currently, the world is facing a geopolitical crisis that is already starting to cause food and energy crises, and people and nations may have to choose between eating and heating. Equally distressing can be the use of food shortages and famine as a political weapon, exemplified by memories of the Holodomor terror-famine and cannibalism in the Ukraine (1932–33) in which 3.5–5 million people died.

The interactions between sociotypes, food security, and culture form a complex adaptive system to advance modern societies towards a One Health, One Planet future. They are inextricably entwined as a three-ply cord to ensure that this can happen. The goal is to eradicate world hunger with the additional hope for fewer conflicts and a more harmonious world. The rest will be history—the continuing cultural history of *Homo culturus*.

## Abbreviations

CAS: complex adaptive system.
